# A pilot study investigating affective forecasting biases with a novel virtual reality-based paradigm

**DOI:** 10.1038/s41598-023-36346-3

**Published:** 2023-06-08

**Authors:** Louise Loisel-Fleuriot, Thomas Fovet, Arnaud Bugnet, Coralie Creupelandt, Marielle Wathelet, Sébastien Szaffarczyk, Stéphane Duhem, Guillaume Vaiva, Mathilde Horn, Fabien D’Hondt

**Affiliations:** 1 Univ. Lille, Inserm, CHU Lille, U1172 - LilNCog - Lille Neuroscience & Cognition, 59000 Lille, France; 2 Department of Psychiatry, CHU Lille, 59000 Lille, France; 3 Centre National de Ressources et de Résilience Lille-Paris (CN2R), 59000 Lille, France; 4 Fédération de Recherche en Psychiatrie et Santé Mentale des Hauts-de-France, 59000 Lille, France; 5 Univ. Lille, Inserm, CHU Lille, CIC1403 - Clinical Investigation Center, 59000 Lille, France; 6 CURE, Service de Psychiatrie de L’enfant et de L’adolescent, Hôpital Fontan 1, CHU de Lille, CS 70001, 59037 Lille cedex, France

**Keywords:** Human behaviour, Emotion, Cognitive neuroscience, Autonomic nervous system

## Abstract

A body of research indicates that people are prone to overestimate the affective impact of future events. Here, we developed a novel experimental paradigm to study these affective forecasting biases under laboratory conditions using subjective (arousal and valence) and autonomic measures (skin conductance responses, SCRs, and heart rate). Thirty participants predicted their emotional responses to 15 unpleasant, 15 neutral, and 15 pleasant scenarios (affective forecasting phase) to which they were then exposed in virtual reality (emotional experience phase). Results showed that participants anticipated more extreme arousal and valence scores than they actually experienced for unpleasant and pleasant scenarios. The emotional experience phase was characterized by classic autonomic patterns, i.e., higher SCRs for emotionally arousing scenarios and greater peak cardiac acceleration for pleasant scenarios. During the affective forecasting phase, we found only a moderate association between arousal scores and SCRs and no valence-dependent modulation of cardiac activity. This paradigm opens up new perspectives for investigating affective forecasting abilities under lab-controlled conditions, notably in psychiatric disorders with anxious anticipations.

## Introduction

Affective forecasting skills refer to human individuals' abilities to predict their emotional feelings in response to a future event^1^. These skills are crucial for goal-directed decision-making, as the anticipation of possible positive or negative consequences of future events helps to make choices that may promote survival, happiness, and well-being^2,3^. However, current evidence indicates that affective forecasting skills are constrained by several biases, i.e., differences between expectations and actual experiences of a given situation^4^.

The most well-documented affective forecasting bias—the "impact bias"—is characterized by the tendency to predict more intense and long-lasting emotional feelings than those that are actually experienced^1^. This bias and its moderators have been explored in longitudinal studies comparing affective forecasts, i.e., the predictions about emotional responses (i.e., the intensity of pleasure/displeasure, or specific emotions such as joy or fear) when evoking a given future situation and the emotions experienced when faced with the same situation in real life^5–7^. Impact bias has mainly been studied for negative events, e.g., unsuccessful track athletes overestimating their negative feelings^8^, but it has also been observed for positive events, e.g., football supporters predicting more intense positive reactions to their favorite football team's victory^9^. However, affective forecasting has never been studied and compared to emotional experience by distinguishing the two most accepted dimensions of emotion, namely, valence, which indexes the level of pleasure/displeasure, and arousal, which indexes the level of intensity^10^. Moreover, these studies, centered on one single given event, do not allow for the exploration of forecast biases for negative and positive situations within the same sample.

Another important limitation of studies that focus on affective forecasting is that they have exclusively used subjective measures, which hampers the full understanding of affective forecasting biases, including their neurocognitive correlates. The literature on emotional experience suggests that several anterior brain regions, notably the amygdala and the ventromedial prefrontal cortex, play a role in assessing the affective significance of stimuli and trigger central and peripheral responses, including modulations of attention and autonomic changes^11–13^. Emotional feelings are then supposed to emerge from the subjective experience of these responses^14,15^. Evidence indicates that subjective arousal scores associated with affectively laden stimuli increase with the amplitude of the skin conductance responses (SCRs) to these stimuli^10^. Given that SCRs reflect sympathetic nervous system responses, they are widely regarded as a reliable autonomic marker of emotional arousal^16^. Although the relationship between heart rate (HR), which depends on the sympathetic and parasympathetic nervous systems, and subjective reports is more complex^10,17^, research has found links between the valence attributed to a stimulus and the variations in HR induced by the same stimulus. More specifically, the peak HR acceleration that follows the initial deceleration induced by visual scenes is positively correlated with the level of pleasure^10^.

The dominant model posits that affective forecasting involves mental simulations of future events, which generate immediate emotional responses that are used to predict the emotions that are likely to occur in response to these events^18^. Of most interest, evidence suggests that anterior brain regions involved in the initial affective assessment of stimuli during emotional experience also participate in affective forecasting^18,19^. If similar affective processes are at play during the mental simulation of future events and the actual experience of these events, then comparable associations between subjective (valence and arousal) and autonomic (cardiac and electrodermal activity) measures may also be assumed during the anticipation of emotional responses to future events.

Here, we developed a novel experimental paradigm to assess and compare arousal and valence responses associated with affective forecasting and actual emotional experience of multiple pleasant, neutral, and unpleasant scenarios using subjective and autonomic measures within the same sample of participants and under lab-controlled conditions. During this two-phase paradigm, participants first have to predict their emotional responses (i.e., arousal, valence) to a set of unpleasant, neutral, and pleasant scenarios ("affective forecasting" phase) and are then exposed to these scenarios using a virtual device ("emotional experience" phase). Following previous results^8,20,21^, we predicted higher arousal scores for unpleasant and pleasant situations but not neutral situations in the affective forecasting phase than in the emotional experience phase and higher and lower valence scores for pleasant and unpleasant situations, respectively, in the affective forecasting phase than in the emotional experience phase. In line with classical observations on emotional experiences^10,17^, we expected a positive association between arousal scores and amplitude of electrodermal responses and thus higher electrodermal responses to unpleasant and pleasant situations compared to neutral situations in the emotional experience phase. We also expected a positive association between valence scores and peak cardiac acceleration, and thus increases in peak cardiac acceleration from unpleasant, neutral, and finally pleasant situations. Following Gilbert & Wilson's model^18^, we hypothesized that similar autonomic results would be obtained during the affective forecasting phase.

## Methods

### Participants

Thirty healthy adults took part in the study (17 females; M = 32.83, SD = 9.8 years; 26 right-handed). Because this study used a new experimental paradigm, we had no available data to determine the appropriate sample size. They all provided informed consent before participating in the study, which was conducted in accordance with the Declaration of Helsinki. The study was approved by the French ethics committee (Comité de protection des personnes Ouest II, Angers).

Participants had normal or corrected-to-normal vision and audition; they did not present any personal history or current neurological diseases, psychiatric disorders or addictions as assessed by the Mini International Neuropsychiatric Interview (MINI)^22^. On average, participants had low (M = 40.9, SD = 6.9) and moderate (M = 46.1, SD = 8.6) scores for state and trait anxiety, respectively, as measured with the State and Trait Anxiety Inventory form Y (STAI-Y)^23^. Participants presented normal to moderate depression symptoms (M = 7.5, SD = 5.3) as assessed with the Beck Depression Inventory (BDI-II)^24^. Participants were recruited by public announcements in the Lille University Hospital, Lille University, and social media and were paid 20 euros for their time.

### Stimuli

We used forty-five scenarios (15 pleasant, 15 unpleasant, and 15 neutral) ranging from everyday scenes to more arousing and uncommon scenes, involving the same number of animals, landscapes, and interindividual interaction scenes per emotional category. These scenarios correspond to 45 360° videos from either the C2Care database (https://www.c2.care/en/) or websites offering free materials (e.g., a bike ride, puppies, spiders). The videos each lasted 20 s. For the affective forecasting phase, descriptions of these videos were built in the form of two short sentences (example: "*You are on the beach. You see the turquoise water near other vacationers."*). An audio version was recorded with a female voice reading the sentences in a neutral tone. Our decision to present information in a multimodal format that engaged participants through both visual and auditory channels was motivated by the potential benefits of multisensory integration, which include increased attention and improved comprehension.

These descriptions were provided to a group of 28 healthy individuals (15 females; M = 25.82, SD = 12.01 years) during a pretest phase in which the participants assessed the arousal [from 1 (very calm) to 9 (very excited)] and the valence [from 1 (very unpleasant) to 9 (very pleasant)] dimensions of the scenarios using the Self-Assessment Manikin (SAM)^25^. The mean values for valence and arousal were 2.56 (SD = 0.62) and 6.70 (SD = 1.28) for the unpleasant stimuli, 5.27 (SD = 0.46) and 2.30 (SD = 0.37) for the neutral stimuli, and 7.76 (SD = 0.36) and 4.53 (SD = 1.36) for the pleasant stimuli, respectively.

### Procedure

Participants were seated comfortably in a chair in front of a computer. They completed the questionnaires, and then, the physiological data collection devices were installed before the start of the experiment.

**Figure 1:**
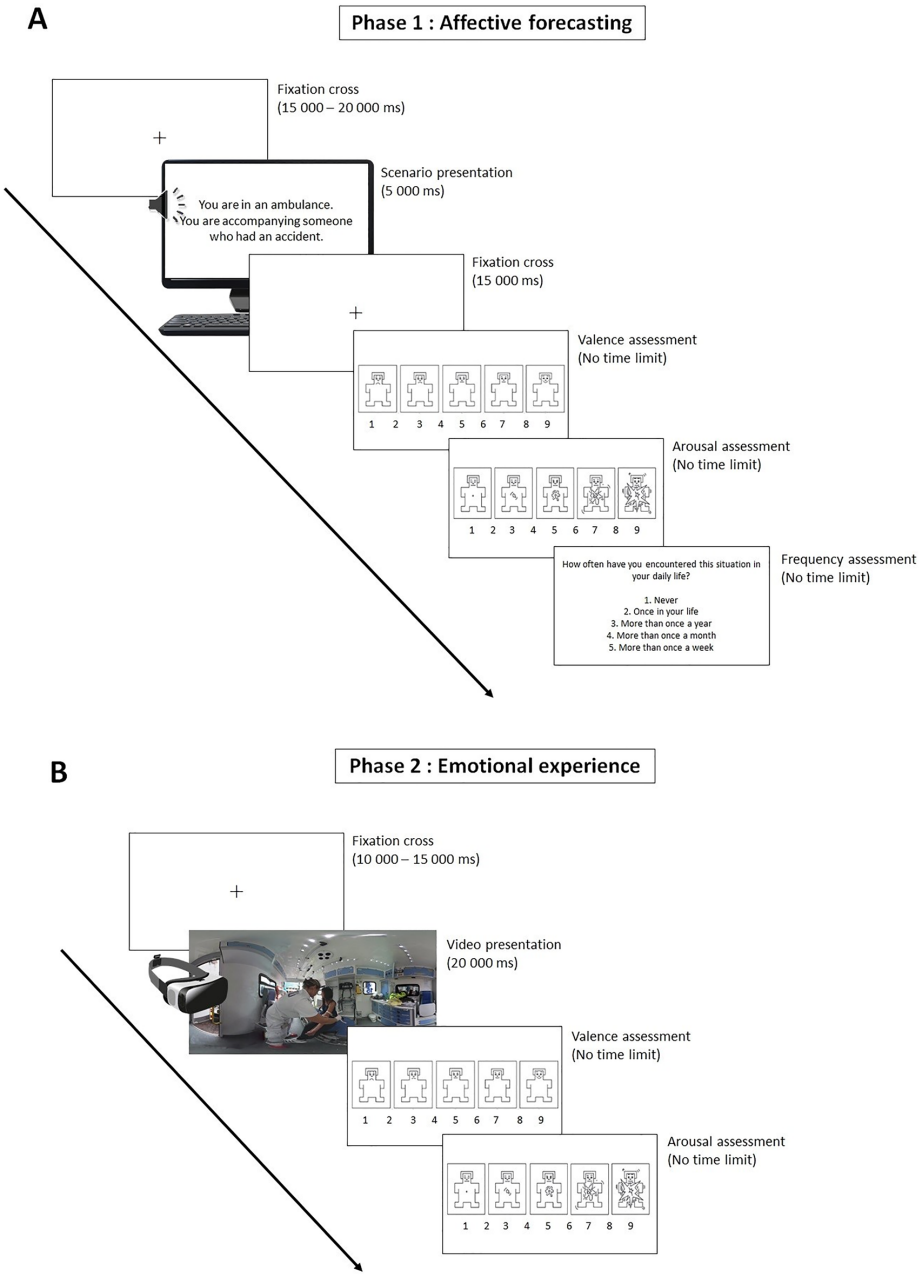
Illustration of the task. (**A**) Affective forecasting phase. Each trial started with a fixation cross, followed by the simultaneous display of sentences describing a scenario on a computer screen and through headphones. A second fixation cross was then presented during which participants predicted their emotional responses to the scenario if they were to experience it. Finally, participants completed the valence and arousal scales of the Self-Assessment Manikin (SAM) and rated the frequency of the scenario in daily life on a Likert scale. (**B**) Emotional experience phase. Each trial started with a fixation cross, followed by the presentation of a scenario on a virtual reality device. Then, participants rated their emotional responses by completing the valence and arousal scales of the SAM.

*Affective forecasting phase.* In the first experimental phase, after the presentation of the instructions, each trial started with a fixation cross (presented randomly between 15 and 20 s) followed by the presentation of a scenario for approximately 5 s (Fig. 1). During the following 15 s, participants were asked to predict their emotional responses to the scenario if they were to experience it (see Supplemental information). Then, they completed the SAM and the frequency scales without a time limit.

We allocated 20 s (5 s for the presentation of the scenario and 15 s for the affective forecasting task) per scenario because it corresponded to the duration of the associated videos. This ensured that participants had an equal amount of time processing scenarios during both the affective forecasting and emotional experience phases of the study. This first experimental phase comprised 45 trials and lasted approximately 45 min. Of note, participants were not informed during this phase that they would virtually experience these same specific scenarios in a second phase.

*Emotional experience phase.* After a few minutes break, the virtual reality device was installed for the emotional experience phase. Each trial started with a fixation cross (presented randomly between 10 to 15 s) followed by a 20-s video. Then, participants completed the SAM without any time limit. This second phase included 45 trials and lasted approximately 30 min.

In both experimental phases, the stimuli were presented in a pseudorandomized order (no more than two scenarios of the same emotional category could appear in a row). Instructions given to the participants can be found in Supplementary information. At the end of the experience phase, participants completed the presence questionnaire in virtual reality, and we collected their feedback and potential questions about the study.

### Experimental setup

#### Affective forecasting phase

Affective forecasting scenarios and questionnaires were presented with the E-prime® software (Psychology Sofware Tools, Inc., Pittsburgh, PA; text size: 44, typeface: Calibri bold, text color: black, background color: gray) visually on a computer screen in sentence form (DELL P2414H, resolution: 1920 × 1080, 60 Hz) and through headphones (Beyerdynamic DT 990 Pro 250 OHM). Participants responded to the SAM and the frequency scale using the number pad on a keyboard. The computer (DELL precision 3650, CPU: i9-10900 K, OS: Windows 10, graphic card: GeForce RTX 3070) was connected to a laptop dedicated for physiological recordings to synchronize the onset of the stimuli and the recording of the autonomic data.

#### Emotional experience phase

For the emotional experience phase, the 360° videos were presented using an Oculus Quest 2 headset version Development Kit 2 (Oculus VR.), which uses an LCD screen with a temporal resolution of 120 Hz and a spatial resolution of 1832 × 1920 pixels per eye (Meta Platforms, Inc, Menlo Park, California, United States). This screen, placed a few centimeters away from the eyes perpendicular to the axis of the gaze, displayed a stereoscopic image and allowed a visual field of approximately 110°. Participants responded to the SAM directly in the virtual interface using a virtual reality controller with their dominant hand. More precisely, they had to target the corresponding number with the virtual reality controller and press a button with the index finger to validate their response. The virtual reality program was coded with Unity® Software (2021.2.1, Unity Technologies, San Francisco, California, United States), launched with the computer used in the affective forecasting phase, and synchronized with the laptop used for physiological data acquisition.

### Data recording and processing

#### Subjective measures

During the two experimental phases, participants had to assess the arousal [from 1 (very calm) to 9 (very excited)] and valence [from 1 (very unpleasant) to 9 (very pleasant)] dimensions of the scenarios with the SAM^25^. Both the affective forecasting and emotional experience phases utilized computerized versions of the scales. During the affective forecasting phase, participants also rated the frequency of the scenarios in daily life on a Likert scale from 1 (never) to 5 (more than once a week).

Participants completed an adapted version of the Immersion Propensity Questionnaire (IPQ) to assess their ease of being immersed and the Presence Questionnaire (PQ) [French version translated by the Cyberpsychology Laboratory of the University of Quebec in Outaouais (2002), original version by Witmer & Singer (1998)^26^] to assess the perceived quality of virtual environments (see Supplementary information).

#### Physiological measures

Physiological signals were acquired with an MP160WSW multichannel integration system coupled with BioNomadix wireless devices to collect electrodermal activity (EDA) and HR data (BIOPAC Systems, Inc.). Data were initially collected at a rate of 2000 Hz. The laptop dedicated to physiological data acquisition (HP ZBook 15 G2, 1000 Hz) displayed the participants' electrodermal and cardiac activity in real-time with Acknowledge® software (Biopac, System) through a connection to the BIOPAC device.

EDA was recorded using 2 disposable Ag/AgCl electrodes with a contact area of 11 mm (Biopac EL507) filled with isotonic gel (0.5% saline in a neutral base, Biopac GEL101) using the Dermal Conductance technique. The electrodes were placed on the palmar surface of the nondominant hand (middle phalanx of the index and middle fingers). EDA signals were resampled offline at 50 Hz and quantified using the Continuous Decomposition Analysis (CDA) method from the Ledalab Toolbox (Version 3) in MATLAB (R2015a, The MathWorks, Inc., Natick, Massachusetts, United States). This method allows the extraction of the phasic (SCRs) and tonic (skin conductance level) components from the electrodermal signal. An analysis of the integrated skin conductance responses (ISCRs) was conducted for each trial and phase for each participant. This indicator, expressed in µS*s, corresponds to the area (i.e., time integral) of the phasic response relative to the analysis window. Adaptative smoothing using the Gaussian method and a low-pass Butterworth filter (first-order low-pass filter with a 5 Hz cutoff) were applied to the data. Following the current recommendations for EDA analysis, the response window extended from 1 to 10 s after stimulus onset, and the minimum threshold of the response amplitude was 0.01 *µS*^27^. Finally, the data were square-root transformed to reduce skewness.

Electrocardiograms (ECGs) were recorded using 3 disposable Ag/AgCl electrodes (Biopac EL503) filled with conductive gel (Biopac GEL100). One electrode was placed on each clavicle and the left lower rib. Each individual’s HR was computed from the ECG with Acknowledge® software (Biopac, System). For each participant and each trial of each phase, we computed the difference between the instantaneous HR and baseline HR (i.e., the average instantaneous HR during the 3 s before stimulus onset). We determined the peak HR acceleration as the maximal value of HR variation that occurred after the minimal HR variation within the first 6 s after stimulus onset.

### Statistical analyses

The final pre-processed dataset is available on OSF (https://osf.io/afntg/). Overall, 122 trials (representing, on average, 1.13%, S.D. = 2.28, for each participant) were discarded from the analyses due to technical issues. The analyses were conducted with R software (4.1.2). We applied linear mixed models (LMM) using the "lmerTest" package^28^ on subjective (arousal and valence scores) and autonomic (ISCR and peak cardiac acceleration) data, with Emotion (unpleasant, neutral, and pleasant) and Experimental phase (affective forecasting and emotional experience) as fixed effects and Participants as a random effect (random intercept). Diagnostics of linearity, homoscedasticity, and normality of residuals and random effects indicated that the model assumptions were met. We applied a Type III analysis of variance with Satterthwaite's method to each LMM to assess the global effects of each predictor, using a threshold of significance at *p < *0.05. In case of significant interactions, we performed followed-up contrasts with the "emmeans" package^29^ by computing t-ratios with the Kenward-Roger's method based on the estimated marginal means from the LMM. We were specifically interested in nine comparisons: we intended to compare emotions in each phase and across phases. For these follow-up contrasts, statistical significance was thus accepted at a Bonferroni-adjusted alpha level of 0.005. For each LMM, we used the "piecewiseSEM" package^30^ to calculate the Marginal and Conditional R^2^, which represent the proportion of variance explained by the fixed effects, and by both the fixed and random effects.

The associations between subjective and autonomic responses in each experimental phase were assessed by calculating Bravais-Pearson correlational coefficients using mean values computed for each scenario (from the forecasting and experience phases). For each association, we conducted a bootstrap analysis (2000 replications) of the Pearson correlation.

## Results

### Subjective measures

#### Arousal

**Figure 2:**
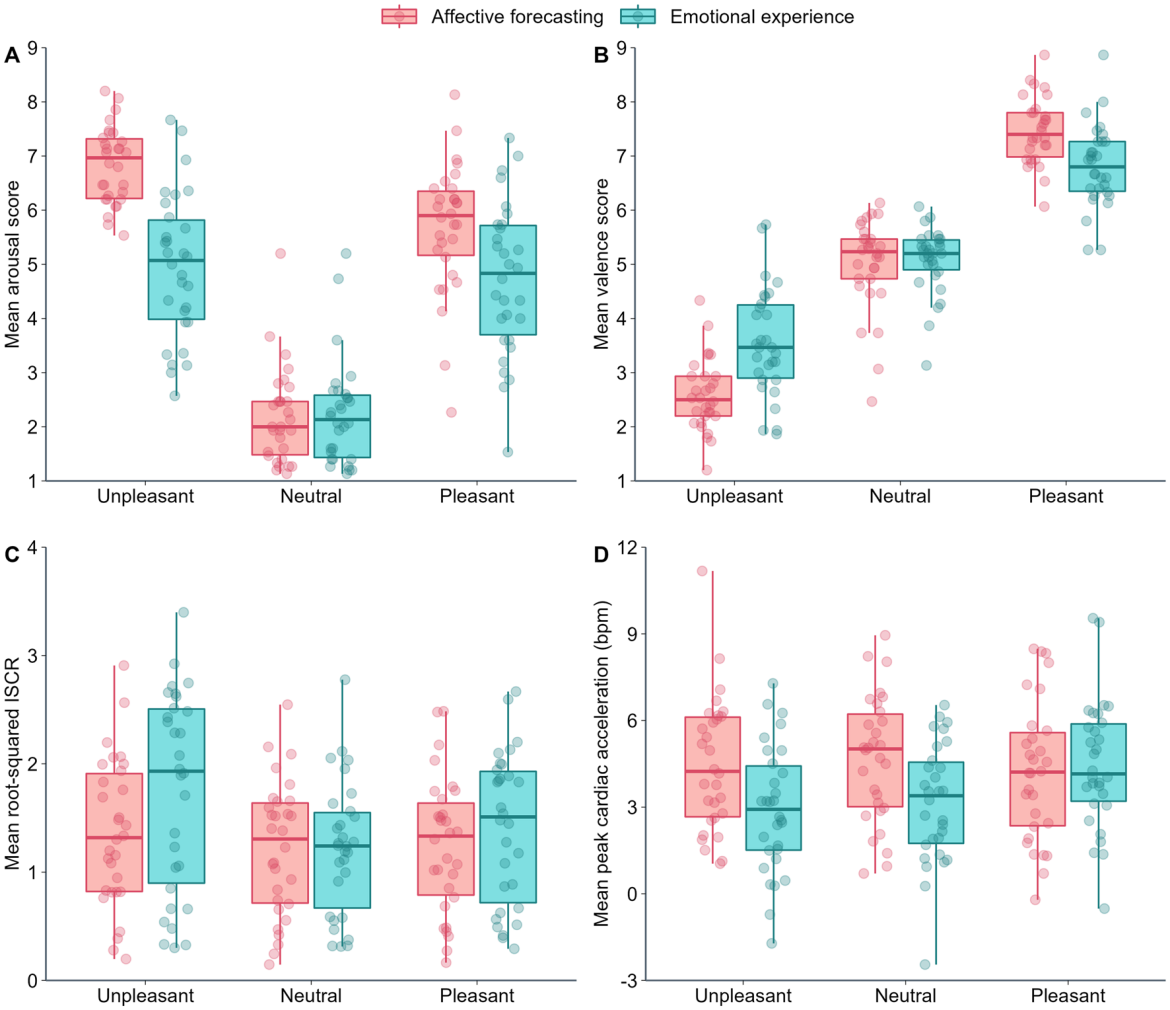
Subjective and autonomic measures according to Emotion (unpleasant, pleasant, neutral) and Experimental phase (affective forecasting, emotional experience). (**A**) mean valence score, (**B**) mean arousal score, (**C**) mean root-squared ISCR (in microsiemens per second), and (**D**) mean cardiac acceleration (in beats per minute).

Marginal and Conditional R^2^ values were 0.42 and 0.49, respectively. There was a significant interaction between Emotion and Phase (F(2,2633) = 54.39, *p < *0.001; Fig. 2A). In the affective forecasting phase, arousal was higher for pleasant than neutral stimuli (t(2633) = 27.77, *p < *0.001), for unpleasant than pleasant stimuli (t(2633) = 8.812, *p < *0.001), and for unpleasant than neutral stimuli (t(2633) = 36.45, *p < *0.001). In the emotional experience phase, arousal was higher for pleasant than for neutral stimuli (t(2633) = 20.02, *p < *0.001), did not differ between unpleasant and pleasant stimuli (t(2633) = 1.79, *p = *0.074), and was higher for unpleasant than for neutral stimuli (t(2633) = 21.70, *p < *0.001). Finally, arousal was higher in the affective forecasting phase than in the emotional experience phase for pleasant (t(2633) = 7.51, *p < *0.001) and unpleasant stimuli (t(2633) = 14.43, *p < *0.001), but did not differ between the phases for neutral stimuli (t(2633) = − 0.23, *p = *0.819).

#### Valence

Marginal and Conditional R^2^ values were 0.52 and 0.55, respectively. There was a significant interaction between Emotion and Phase (F(2,2651) = 59.72, *p < *0.001; Fig. 2B). In the affective forecasting phase, valence was higher for pleasant than neutral stimuli (t(2651) = 23.27, *p < *0.001), for pleasant than unpleasant stimuli (t(2651) = 46.28, *p < *0.001), and for neutral than unpleasant stimuli (t(2651) = 23.07, *p < *0.001). In the emotional experience phase, valence was higher for pleasant than neutral stimuli (t(2651) = 16.44, *p < *0.001), for pleasant than unpleasant stimuli (t(2651) = 30.86, *p < *0.001), and for neutral than unpleasant stimuli (t(2651) = 14.46, *p < *0.001). Moreover, valence was higher in the affective forecasting phase than in the emotional experience phase for pleasant stimuli (t(2651) = 6.18, *p < *0.001), lower in the affective forecasting phase than in the emotional experience phase for unpleasant stimuli (t(2651) = − 9.24, *p < *0.001), and did not differ between phases for neutral stimuli (t(2651) = − 0.65, *p = *0.513).

### Autonomic measures

#### Integrated skin conductance responses (ISCRs)

Marginal and Conditional R^2^ values were 0.02 and 0.36, respectively. There was a significant interaction between Emotion and Phase (F(2,2629) = 7.82, *p < *0.001; Fig. 2C). In the affective forecasting phase, ISCRs were higher for unpleasant than for neutral (t(2629) = 2.50, *p = *0.013) and pleasant stimuli (t(2629) = 1.96, *p = *0.0497), but both differences did not reach statistical significance at a Bonferroni-corrected alpha level of 0.005. ISCRs did not differ between neutral and pleasant stimuli (t(2629) = − 0.54, *p = *0.588). In the emotional experience phase, ISCRs were higher for unpleasant than for pleasant (t(2629) = 5.72, *p < *0.001) and neutral stimuli (t(2629) = 7.97, *p < *0.001). ISCRs were also higher for pleasant than neutral stimuli (t(2629) = 2.27, *p = *0.023), but this difference did not reach statistical significance at a Bonferroni-corrected alpha level of 0.005. Moreover, ISCRs were lower in the affective forecasting phase than the emotional experience phase for unpleasant stimuli (t(2629) = − 5.43, *p < *0.001), but did not significantly differ between phases for pleasant (t(2629) =  − 1.71, *p = *0.088), and neutral stimuli (t(2629) = 0.02, *p = *0.982).

#### Peak cardiac acceleration

**Figure 3:**
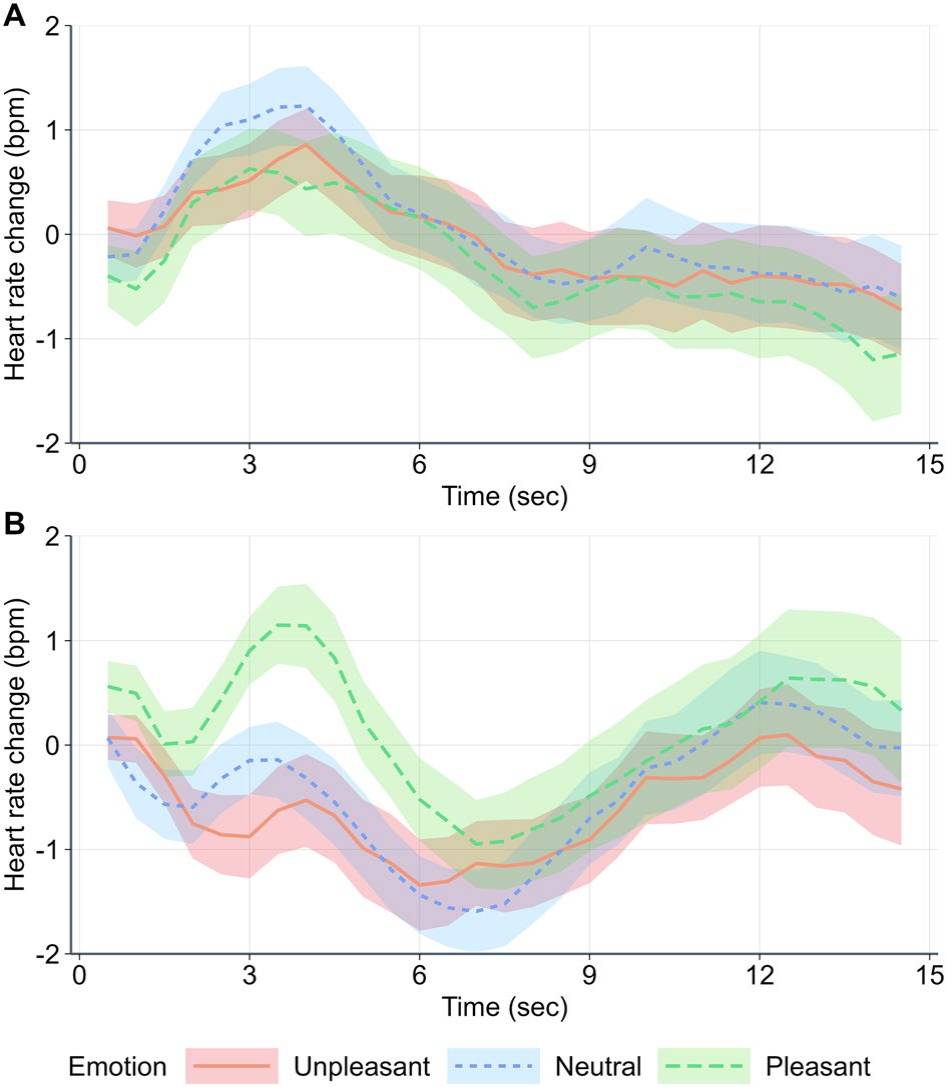
Grand average of heart rate changes (in beats per minute) over time (in seconds), depending on Emotion (unpleasant, pleasant, neutral) and Experimental phase: (**A**) affective forecasting and (**B**) emotional experience. The ribbons represent standard error (SE).

Figure 3 illustrates heart rate changes over time depending on Emotion and Experimental phase. Marginal and Conditional R^2^ values were 0.01 and 0.08, respectively. There was a significant interaction between Emotion and Phase (F(2,2625) = 6.96, *p < *0.001; Fig. 2D). In the affective forecasting phase, peak acceleration did not differ between unpleasant and neutral stimuli (t(2625) = -0.55, *p = *0.584), unpleasant and pleasant stimuli (t(2625) = 0.60, *p = *0.550), and pleasant and neutral stimuli (t(2625) = -1.15, *p = *0.250). In the emotional experience phase, peak cardiac acceleration was greater for pleasant than for neutral stimuli (t(2625) = 3.39, *p < *0.001) and unpleasant stimuli (t(2625) = 4.00, *p < *0.001), and did not differ between neutral and unpleasant stimuli (t(2625) = 0.62, *p = *0.535). Moreover, the peak acceleration was greater in the affective forecasting phase than the emotional experience phase for unpleasant stimuli (t(2625) = 4.05, *p < *0.001) and neutral stimuli (t(2625) = 4.00, *p < *0.001), but did not significantly differ between phases for pleasant stimuli (t(2625) =  − 0.54, *p = *0.591).

### Correlational analyses

The results of the bootstrapped correlational analyses are described in Table 1. For affective forecasting scenarios, the correlation was significant between the arousal score and ISCR (r = 0.32), and for emotional experience scenarios, between the arousal score and ISCR (r = 0.44) and between the valence score and peak cardiac acceleration (r = 0.38).

**Table 1:** Results of bootstrapped bivariate Bravais-Pearson correlational analyses between subjective and physiological responses within experimental phases.

	R [95% CI]	Bias	Standard error
Affective forecasting scenarios			
Arousal score and ISCR	.32* [.08–.58]	−.02	0.13
Valence score and peak cardiac acceleration	−.16 [-.46–.13]	.00	0.15
Emotional experience scenarios			
Arousal score and ISCR	.44** [.21–.70]	−.02	0.12
Valence score and peak cardiac acceleration	.38** [.13–.64]	−-.00	0.13

*p < .05, **p < .01, ***p < .001.

## Discussion

This study analyzed the subjective (valence and arousal) and autonomic (cardiac acceleration and skin conductance) responses of thirty healthy individuals who had to predict their emotional feelings to possible unpleasant, pleasant, and neutral future events and then experience the scenarios using a virtual reality device. The main results showed that participants anticipated more arousing and more negative/positive feelings than those they actually felt once exposed to unpleasant and pleasant events. In addition, we replicated the classic autonomic patterns during the emotional experience phase, with higher peak cardiac acceleration for pleasant events and greater SCRs for emotionally arousing events. During the affective forecasting phase, we observed a moderate association between arousal scores and SCRs and found no valence-dependent modulation of cardiac activity.

As expected, participants considered the unpleasant and pleasant scenarios to be more arousing than the neutral scenarios and attributed low, intermediate, and high valence scores to unpleasant, neutral, and pleasant stimuli, regardless of the experimental phase. They predicted overly extreme arousal and valence scores for unpleasant and pleasant scenarios but not neutral scenarios during the affective forecasting phase compared to the emotional experience phase, and thus anticipated more arousing and more positive and negative emotions than those they actually felt afterward. Our novel experimental paradigm thereby showed the presence of an impact bias that was not restricted to an overestimation of arousal but also concerned the level of valence^1^ of both pleasant and unpleasant events within the same sample of participants for the first time^8,20,21^. The paradigm was designed to have the experience phase occur shortly after the affective forecasting phase. One might argue that the dampened subjective ratings during the experience phase were due to habituation induced by the repetition of stimuli. However, the stimuli in each phase were not identical per se, as they were related to the same scenarios but presented differently: the affective forecasting phase involved short descriptions, while the emotional experience phase used videos. Furthermore, autonomic results contradicted the hypothesis of habituation from the affective forecasting phase to the emotional experience phase.

The literature on emotional experience indicates that affectively-laden stimuli induce greater sympathetic responses than neutral stimuli^10,12,16,17^. In our study, we found that unpleasant scenarios elicited significantly higher ISCRs than pleasant and neutral scenarios in the emotional experience phase. These differences did not reach statistical significance at the Bonferroni-corrected alpha level in the affective forecasting phase. Furthermore, when comparing the two phases, we observed lower ISCRs to unpleasant stimuli during the affective forecasting phase, which supports the intuitive idea that bodily responses are more pronounced when we actually experience an arousing event than when we simulate it. Results also indicated that regardless of the emotional category of the scenarios, arousal scores and ISCRs were positively correlated in the emotional experience phase, indicating that the greater the sympathetic response, the greater the subjective arousal attributed to the scenario. Interestingly, this association, while somewhat weaker, was also observed during the affective forecasting phase. Our study thus showed that this well-documented link characterizing emotional experience^10^ also applies to affective forecasting. This autonomic-subjective association is in accordance with Gilbert & Wilson’s^18^ model of affective forecasting postulating that mental simulations of future events generate affective reactions that are used to predict the likely emotional consequences of the events. In this context, subjective predictions may be associated with the increase in central and peripheral arousal induced by the mental simulation of a future event, as understood in the case of "real exposure" to an event^14,15^.

Analyses of HR variations revealed that the peak cardiac acceleration was greater for pleasant stimuli than neutral or unpleasant stimuli during the emotional experience phase, which again agrees with previous studies^10,17^. However, cardiac acceleration did not differ among the three emotional categories of stimuli in the affective forecasting phase and was as high as that observed for pleasant stimuli in the emotional experience phase. Following the same logic as that for SCRs, we can expect the absence of emotion-related heart rate modulation during affective forecasting to be due to reduced reactions under simulated experiences compared to real-life experiences. Recent literature has investigated the brain processes associated with the anticipation of upcoming affective stimuli and found valence-sensitive neural responses depending on stimuli predictability^31,32^. In our study, the instruction for the affective forecasting phase did not emphasize that participants would virtually experience, in a distinct second phase, the scenarios they were about to simulate. As a result, one could assume that the valence-related neurophysiological changes associated with emotional predictions in cue-event paradigms are linked to the anticipation of the upcoming affective event rather than to the mental simulation of this event. Consistent with this idea, research has shown that following an initial deceleration, heart rate increases in response to cues indicating the need for rapid action to secure a reward or prevent a negative outcome^33^. Moreover, this acceleration is more pronounced in response to positive cues than to neutral or negative ones. Furthermore, correlations between valence scores and cardiac acceleration appeared to be significant during the emotional experience phase, as previously reported in the literature^17^, but not during the affective forecasting phase. In other words, there was no emotion-related modulation of HR acceleration during the affective forecasting phase, and the level of valence predicted for hypothetical future events was not positively correlated with cardiac acceleration during the mental simulation of these events. This result questions the sequence of processing that leads to predicted feelings, as it goes against the idea that the level of pleasantness/unpleasantness predicted for a future event is associated with the body responses induced by the mental simulation of the event, including the early phase of affective processing^11^.

Taken together, our results indicate that the affective assessment of a simulated event is not entirely comparable to that of an experienced event and that affective forecasting biases cannot only be explained by emotional responses induced by the mental simulation of future events. The physiological results indicate that affective forecasting for hypothetical future events is associated with moderate autonomic responses. The ISCR results showed low sympathetic reactivity. Because HR acceleration can be caused by direct sympathetic activation or vagal release^34^, HR results may also indicate no parasympathetic contribution as a function of valence. The temporal distance between affective forecasting and the predicted event may play a critical role, as more pronounced autonomic responses are typically associated with situations in which the body needs to prepare for action^35^. Therefore, further studies should investigate whether affective forecasting of imminent events promotes response mobilization and whether this is associated with the triggering of specific autonomic patterns that vary depending on emotional arousal and valence.

Affective forecasting bias could be understood as a cognitive bias with a cardinal motivational dimension: imagining future events as more unpleasant or pleasant than they will actually be may help increase proactive behaviors to reduce or increase the probability of being confronted with their consequences^3,5^. Another possibility is that affective forecasting bias is determined by an individual’s ability to simulate a future event with precision and that this ability is related to the frequency with which the individual has been confronted with the event or a similar event in the past. Affective forecasting would therefore be linked to our ability to recruit our episodic memories^36–38^ due to the activation of memory regions associated with episodic future thinking^18,39^. To test this hypothesis, we performed supplementary analyses based on the frequency scores associated with each scenario (see Supplementary information). We found that the more frequent an event in everyday life, the less important the affective forecasting bias in terms of arousal. Because unpleasant and pleasant scenarios were also considered to be less frequent than neutral scenarios, we performed correlational analyses between event frequency and affective forecasting bias for each subset of pictures according to their emotional category. The correlations were not significant but remained moderate for emotional stimuli, while they were close to 0 for neutral stimuli. Although preliminary, these results lend support to the notion that our past experiences play a moderating role in affective forecasting biases. This idea is also supported by the recent work by Del Popolo Cristaldi et al.^40^ that employed a cue-event paradigm to investigate how experiencing previous certain versus uncertain contingencies shaped subjective reactions to future affective stimuli. The results indicated that experiencing previous reliable associations leads to subsequently predicting similar associations. However, studies on affective forecasting tend to show that the prediction of our emotional feelings about a future event (e.g., going to the dentist) is more influenced by the most salient experience we have had in the past (such as receiving treatment for a cavity), rather than our typical experiences (such as a routine scaling)^18^. Therefore, further research is needed to clarify the relative contribution and possible interaction between the predictability of stimuli and the salience of past experiences.

This study has limitations. While the use of virtual reality allowed lab conditions, improving control over previous studies^1^, and while it is reassuring that the arousal and valence scores were significantly correlated between the two experimental phases (see Supplementary information), the downsides of this experimental context were probably lower ecological validity of the emotional experience phase^19^ and/or influences on the efficiency of mental stimulation skills. However, we found no significant association between the ease of participants to be immersed as assessed by IPQ scores and affective forecasting biases. We did not find any significant link between the perceived quality of virtual environments as assessed by PQ scores and subjective and autonomic responses to emotional experience scenarios neither (see Supplementary information). In addition, although we enlarged the range of scenarios, their arbitrary-chosen fixed display duration may also have contributed to lowering the ecological validity. Finally, although our exploratory study yielded promising results, it is important to acknowledge that our sample size was limited to only thirty participants. Consequently, the generalizability of our findings may be somewhat constrained. Specifically, the absence of significant findings regarding ISCR to pleasant scenarios could be attributed to the stimuli not being sufficiently arousing or partly due to the small sample size. Nevertheless, our pilot data can serve as a valuable resource for estimating effect sizes and determining appropriate sample sizes in future replication studies.

Despite these limits, our experimental paradigm allowed the well-described impact bias to be reproduced and opens compelling prospects for improvement, among which the possibility to control for additional key factors such as episodic memory—as suggested by our correlational results considering event frequency —or to estimate when a future event will occur and the impact of the time-lapse between the forecast and experience^41,42^. The ease of use and time efficiency of this laboratory paradigm also makes it suitable for research in psychiatric conditions associated with affective biases, such as depression and anxiety^7,43–45]^, and potentially all emotion-dysregulation disorders. Indeed, maladaptive coping strategies, such as the avoidance of any aversive situation, could result from affective forecasting biases, especially since the latter may be partly explained by an underestimation of one’s ability to regulate his or her emotions in the face of a future aversive event^1,9^.

To conclude, to the best of our knowledge, this study was the first to explore the subjective and autonomic correlates of affective forecasting biases associated with multiple scenarios within the same sample in laboratory conditions. The results obtained with the new paradigm assessing the arousal and valence dimensions of emotions refined our understanding of the impact bias, by showing that participants predicted more arousing and more pleasant/unpleasant emotions to future events than what they actually experienced when exposed to these events. Our results partially support Gilbert and Wilson’s^18^ model by showing links between subjective predictions of arousal and autonomic responses to forecasted emotional events. This suggests that affective forecasting biases probably involve additional factors associated with mental simulation, whose neurocognitive determinants will need to be appraised in future studies. Finally, and importantly, this novel experimental paradigm opens new interesting perspectives to study, in lab-controlled conditions, affective forecasting biases in clinical populations, and in particular in psychiatric disorders in which individuals appear to have strong anxious anticipations.

## Supplementary Information

Supplementary Information 1.

Supplementary Information 2.

## Data Availability

The dataset generated during the current study is available on OSF at the link: https://osf.io/afntg/.
