# Supplementary material for: A pilot study investigating affective forecasting biases with a novel virtual reality-based paradigm
Source: Sci Rep. 2023 Jun 8;13:9321. doi: 10.1038/s41598-023-36346-3 (PMC10250404; doi:10.1038/s41598-023-36346-3)
Supplement: Supplementary file 1 — Supplementary Information 1. [file 41598_2023_36346_MOESM1_ESM.docx]

A novel virtual reality-based paradigm for investigating affective forecasting biases

Louise **Loisel-Fleuriot**^1^, Thomas **Fovet**^1,2^, Arnaud **Bugnet**^1^, Coralie **Creupelandt**^3^, Marielle **Wathelet**^1,2,3,4^, Sébastien **Szaffarczyk**^1^, Stéphane **Duhem**^2,3,4,5^, Guillaume **Vaiva**^1,2,3^, Mathilde **Horn**^1,2^, & Fabien **D'Hondt**^1,2,3,^*

*^1^ Univ. Lille, Inserm, CHU Lille, U1172 - LilNCog - Lille Neuroscience & Cognition, F-59000 Lille, France*

*^2^ Department of Psychiatry, CHU Lille, F-59000 Lille, France*

*^3^ Centre national de ressources et de résilience Lille-Paris (CN2R), F-59000 Lille, France*

*^4^ Fédération de Recherche en Psychiatrie et Santé Mentale des Hauts-de-France, F-59000 Lille, France*

*^5^ Univ. Lille, Inserm, CHU Lille, CIC1403 - Clinical Investigation Center, F-59000 Lille, France*

# **Supplementary Methods**

**Instructions**

Affective forecasting phase:

*“In this first phase of the experiment, short scenarios will be presented to you in both oral and written form (i.e., they will be simultaneously displayed on the screen and audible through the headphones). These scenarios describe life situations (e.g., "You are in a living room. You are watching TV."). Even if some situations seem unlikely, we ask that you answer all the questions as SPONTANEOUSLY as possible. You need to determine what your EMOTIONS would be if you were confronted with the situation described. Specifically, for each scenario, you will be asked to answer the following questions (you have approximately 20 seconds to think about it after the situation is described). There are no right or wrong answers”.*

Emotional experience phase:

*In this second phase of the experiment, you will experience different situations through a virtual reality device. You will be able to move your head to explore the environment, but the rest of your body will have to remain still. For each situation, you will be immersed for approximately 20 seconds. Then, you will be asked to answer two questions about your emotional feelings in response to these environments.*

**Correlational analyses between phases for each subjective and autonomic measure**

For each subjective and autonomic measure, we examined the relationship between the two phases by calculating Bravais-Pearson correlational coefficients using mean values computed for each scenario. For each association, we conducted a bootstrap analysis (2000 replications) of the Pearson correlation.

**Frequency, immersion, and presence measures**

Frequency scores were analyzed with a linear mixed model (LMM) with the emotion (unpleasant, neutral, and pleasant emotions) defined as fixed effects and participants as random effects (random intercept adjusted for each participant). We used the Type III analysis of variance with Satterthwaite's method to measure the global effect of factors. In case of significant interactions, we performed followed-up contrasts with the "emmeans" package (Lenth et al., 2023) by computing t-ratios with the Kenward-Roger's method based on the estimated marginal means from the LMM. We were specifically interested in nine comparisons: we intended to compare emotions in each phase and across phases. For these follow-up contrasts, statistical significance was thus accepted at a Bonferroni-adjusted alpha level of 0.005. We used the "piecewiseSEM" package (Lefcheck, 2016) to calculate the marginal and conditional R², which represent the proportion of variance explained by the fixed effects, and by both the fixed and random effects.

Moreover, bootstrapped bivariate Bravais-Pearson correlations (2000 replications) were analyzed between frequency scores and subjective biases (calculated as the difference between the mean values of the scores for each pair of scenarios between the forecast and experience phases) across all scenarios, and each emotional category (unpleasant, neutral, pleasant) was considered separately. We conducted similar bootstrapped correlation analyses between immersion scores and forecasting biases for each subjective and autonomic measure and between presence scores and subjective and autonomic responses during the emotional experience phase.

## **Supplementary Results**

**Frequency**

Marginal and Conditional R² values were 0.47 and 0.49, respectively. There was a main effect of the emotion (F(2,1312.1) = 614.65, p < .001). Unpleasant stimuli were judged as being less frequent than pleasant (t(1312) = -12.61, p = < .001), and neutral stimuli (t(1312) = -34.62, p < .001). Pleasant stimuli were judged as being less frequent than neutral stimuli (t(1312) = -22.08, p < .001).

The results of the bootstrapped correlational analyses are described in Supplementary Table 1. Correlations between frequency scores and subjective arousal biases were significant across all scenarios (r = -.77), but there were no other significant correlations.

**Immersion and presence**

The results of the bootstrapped correlational analyses are described in Supplementary Table 1. There was no significant correlation between immersion scores and forecasting biases for any subjective and autonomic measure or between presence scores and subjective and autonomic responses during the emotional experience phase.

**Correlational analyses between phases for each subjective and autonomic measure**

The results of the bootstrapped correlational analyses are described in Supplementary Table 2. Analyses also revealed significant correlations between phases for the arousal score (r = .94), valence score (r = .95), and ISCR (r = .73).

## **Supplementary Table 1**

Results of bootstrapped bivariate Bravais-Pearson correlational analyses between presence, immersion, and frequency scores and subjective and autonomic responses

| AB measures | R [95% CI] | Bias | Standard error |
| --- | --- | --- | --- |
| *Immersion and valence scores for Affective forecasting biases* | | | |
| Unpleasant scenarios | .15 [-.16–.47] | -.01 | 0.16 |
| Neutral scenarios | 07 [-.20–.32] | .01 | 0.13 |
| Pleasant scenarios | -.21 [-.48–.06] | -.01 | 0.14 |
| *Immersion and arousal scores for Affective forecasting biases* | | | |
| Unpleasant scenarios | -.00 [-.35–.34] | .00 | 0.18 |
| Neutral scenarios | -.02 [-.33–.30] | -.00 | 0.16 |
| Pleasant scenarios | -.10 [-.43–.22] | .00 | 0.17 |
| *Immersion and ISCRs for Affective forecasting biases* | | | |
| Unpleasant scenarios | .06 [-.26–.40] | -.01 | 0.17 |
| Neutral scenarios | .06 [-.26–.39] | .00 | 0.17 |
| Pleasant scenarios | -.01 [-.37–.35] | .00 | 0.18 |
| *Immersion and cardiac acceleration for Affective forecasting biases* | | | |
| Unpleasant scenarios | -.04 [-.38–.30] | .00 | 0.17 |
| Neutral scenarios | -.05 [-.39–.30] | .00 | 0.18 |
| Pleasant scenarios | -.06 [-.39–.29] | -.01 | 0.17 |
|  |  |  |  |
| *Presence and valence scores for Emotional experience scenarios* | | | |
| Unpleasant scenarios | -.21 [-.63–.23] | -.01 | 0.22 |
| Neutral scenarios | .06 [-.31–.42] | .01 | 0.19 |
| Pleasant scenarios | .26 [.01–.51] | -.00 | 0.13 |
| *Presence and arousal scores for Emotional experience scenarios* | | | |
| Unpleasant scenarios | .00 [-.39–.38] | .01 | 0.19 |
| Neutral scenarios | .06 [-.29–.47] | -.03 | 0.19 |
| Pleasant scenarios | .10 [-.02–.42] | -.01 | 0.16 |
| *Presence and ISCRs for Emotional experience scenarios* | | | |
| Unpleasant scenarios | .08 [-.25–.44] | -.02 | 0.18 |
| Neutral scenarios | .15 [-.14–.46] | -.01 | 0.15 |
| Pleasant scenarios | .16 [-.16–.50] | -.01 | 0.17 |
| *Presence and cardiac acceleration for Emotional experience scenarios* | | | |
| Unpleasant scenarios | .03 [-.30–.37] | .00 | 0.17 |
| Neutral scenarios | .04 [-.30–.36] | .01 | 0.17 |
| Pleasant scenarios | -.06 [-.45–.31] | .01 | 0.19 |
|  |  |  |  |
| *Frequency and valence scores for Affective forecasting biases* | | | |
| All scenarios | .25 [.00-.50] | .00 | 0.13 |
| Unpleasant scenarios | .02 [-.67–.64] | .04 | 0.34 |
| Neutral scenarios | -.20 [-.51–.13] | -.01 | 0.16 |
| Pleasant scenarios | -.12 [-.64–.39] | .01 | 0.26 |
| *Frequency and arousal scores for Affective forecasting biases* | | | |
| All scenarios | -.77*** [-.85- -.68] | -.00 | 0.05 |
| Unpleasant scenarios | -.30 [-.70–.15] | -.02 | 0.21 |
| Neutral scenarios  Pleasant scenarios | .02 [-.40–.43]  -.51 [-.89–.15] | -.00  .01 | 0.21  0.19 |

Note: *** p < .001

## **Supplementary Table 2**

Results of bootstrapped bivariate Bravais-Pearson correlational analyses between experimental phases for subjective and autonomic measures

|  | R [95% CI] | Bias | Standard error |
| --- | --- | --- | --- |
| Arousal score | .94*** [.91–.98] | -.00 | 0.02 |
| Valence score | .95*** [.91–.98] | -.00 | 0.02 |
| ISCR | .73*** [.50–1.00] | -.02 | 0.13 |
| Cardiac acceleration | .05 [-.23–.33] | .00 | 0.14 |

Note: * p < .05, ** p < .01, *** p < .001
