# Supplementary material for: A pilot study investigating affective forecasting biases with a novel virtual reality-based paradigm
Source: Sci Rep. 2023 Jun 8;13:9321. doi: 10.1038/s41598-023-36346-3 (PMC10250404; doi:10.1038/s41598-023-36346-3)
Supplement: Supplementary file 2 — Supplementary Information 2. [file 41598_2023_36346_MOESM2_ESM.docx]

# **Supplementary information**

Supplementary methods: the presence, immersion, and frequency questionnaire and the instructions delivered to the participants; correlational analyses between phases for each subjective and autonomic measure.

Supplementary results and tables: Analysis of variance on frequency and results of bootstrapped bivariate Bravais-Pearson correlational analyses between: presence, immersion, and frequency scores and subjective and autonomic responses; phases for each subjective and autonomic measure.
